# Nonsymbolic Numerosity Maps at the Occipitotemporal Cortex Respond to Symbolic Numbers

**DOI:** 10.1523/JNEUROSCI.0687-22.2023

**Published:** 2023-04-19

**Authors:** Yuxuan Cai, Shir Hofstetter, Serge O. Dumoulin

**Affiliations:** ^1^School of Psychology, South China Normal University, 510631, Guangzhou, Guangdong, China; ^2^Guangdong Provincial Key Laboratory of Mental Health and Cognitive Science, South China Normal University, 510631, Guangzhou, Guangdong, China; ^3^Spinoza Centre for Neuroimaging, 1105 BK, Amsterdam, Netherlands; ^4^Computational Cognitive Neuroscience and Neuroimaging, Netherlands Institute for Neuroscience, 1105 BA, Amsterdam, Netherlands; ^5^Experimental and Applied Psychology, Vrije University Amsterdam, 1081 HV, Amsterdam, Netherlands; ^6^Experimental Psychology, Helmholtz Institute, Utrecht University, 3584 CS, Utrecht, Netherlands

**Keywords:** numerosity map, symbolic number, ultra-high field fMRI, ventral temporal cortex

## Abstract

Numerosity, the set size of a group of items, helps guide human and animals' behavior and decisions. Numerosity perception is thought to be a precursor of symbolic numerical cognition. Previously, we uncovered neural populations selectively tuned to numerosities organized in a network of topographic maps in human association cortex. Here we investigate whether these numerosity maps are also involved in the processing of symbolic numbers, using 7T fMRI and a number-detection task. We recruited 7 participants (3 females) and found that the numerosity map at the temporal-occipital cortex (NTO) also responds to symbolic numbers. Furthermore, we found that numerosity-tuned neuronal populations at the NTO map in the left hemisphere are tuned to symbolic numbers. These results reveal different functions of the numerosity maps and support a link between numerosity representation and symbolic number processing in the ventral temporal-occipital cortex.

**SIGNIFICANCE STATEMENT** Humans and other animals share an intuitive “number sense” to approximately represent numerosity. However, humans possess a unique ability to process number symbols (e.g., Arabic numbers). It has been argued that the human understanding of symbolic numbers is rooted in our ability to numerosity perception. Here we investigate whether numerosity-tuned neuronal populations organized at a network of topographic maps also respond to symbolic numbers. We find one of the maps at the temporal-occipital cortex is involved in symbolic numerical cognition and the neuronal populations are tuned to numbers. These results provide evidence for a link between nonsymbolic numerosity and symbolic number processing.

## Introduction

Numerosity (i.e., the set size of a group of items) helps guide humans and animals' behavior and decisions (Dehaene, 2001; Nieder, 2020a, 2021). Humans share the ability to perceive numerosity with many animal species, including nonhuman primates (Brannon and Terrace, 1998; Sawamura et al., 2002; Cantlon and Brannon, 2006), birds (Emmerton et al., 1997; Ditz and Nieder, 2015), fish (Agrillo et al., 2008), and insects (Cantlon et al., 2009; Giurfa, 2019). Newborn infants and preverbal infants are also able to perceive nonsymbolic numerosity (Strauss and Curtis, 1981; Feigenson et al., 2004; Izard et al., 2009). However, only human adults possess a unique numerical competence (i.e., symbolic numerical cognition) that involves the learning of abstract symbols, such as Arabic numerals, number words, math, and so forth (Ansari, 2008; Nieder and Dehaene, 2009).

Whether nonsymbolic numerosity and symbolic numbers are represented in a common abstract coding scheme is a longstanding debate (Dehaene, 1992; Nieder, 2004; Ansari et al., 2007; Cohen Kadosh et al., 2007; Piazza et al., 2007). Two competing hypotheses have been proposed. Based on behavioral observations, some researchers propose the existence of two independent numerical systems: one for approximate nonsymbolic numerosities and another for exact symbolic numbers (Sasanguie et al., 2017; He et al., 2021; Marinova et al., 2021). In agreement with this view, neuroimaging evidence has shown distinct neural activation patterns evoked by nonsymbolic and symbolic number formats (Eger et al., 2009; Bulthé et al., 2014). More recently, single-cell recordings in the medial temporal lobe of neurosurgical patients revealed distinct neurons selectively tuned to nonsymbolic and symbolic numbers (Kutter et al., 2018).

Alternatively, another view suggests that nonsymbolic numerosity and symbolic numbers are interconnected. The approximate number system shared by human adults, infants, and animals is believed to be the precursor to the development of symbolic numbers (Dehaene, 2001; Feigenson et al., 2004; Ansari, 2008; Piazza, 2010; Nieder, 2020b). It has often been assumed that number symbols acquire their meaning by being mapped onto the preexisting nonsymbolic representations of numerical magnitude (i.e., the “mental number line”) (Verguts and Fias, 2004). The approximate number system is characterized by two behavioral characteristics: the “numerical distance effect” and “numerical size effect” (Dehaene et al., 1998). Psychophysics studies have demonstrated that both nonsymbolic (Buckley and Gillman, 1974) and symbolic (Moyer and Landauer, 1967) numerical magnitudes are subject to these two effects (Defever et al., 2011). Moreover, and crucially, performance with nonsymbolic numerical tasks predicted children's mathematics performance (Halberda et al., 2008; Gilmore et al., 2010), and training on nonsymbolic arithmetic skills improved symbolic math performance (Park et al., 2016). Brain imaging studies identified regions primarily in the parietal and frontal lobes as key areas of both nonsymbolic and symbolic number processing (Piazza et al., 2007; Arsalidou and Taylor, 2011; Sokolowski et al., 2017). Examination on brain-damaged patients associated deficits at key regions responsible for numerosity processing with dyscalculia and acalculia, a learning disability in comprehending and manipulating numbers (Dehaene et al., 2004).

In the last decades, evidence from single-cell recording in nonhuman primates (Nieder et al., 2002), crows (Ditz and Nieder, 2015), and human (Kutter et al., 2018) have shown neurons tuned to numerosity, responding maximally when a specific numerosity is displayed, with responses decreasing as distance from this preferred numerosity increases. Similar numerosity-tuned responses were shown using an fMRI adaptation paradigm (Piazza et al., 2004; Jacob and Nieder, 2009). We have since used population receptive field (pRF) modeling (Dumoulin and Wandell, 2008) to show that these neural population responding to specific numerosities are organized in topographic maps where preferred numerosity changes gradually across the cortical surface (Harvey et al., 2013). A network of these numerosity maps were found throughout the human cortex, specifically in the temporal-occipital lobe (NTO), parietal-occipital lobe (NPO), parietal lobe (NPC1-3), and frontal lobe (NF) (Harvey and Dumoulin, 2017; Cai et al., 2021b; Hofstetter et al., 2021; Tsouli et al., 2021). However, the role these maps play in numerosity perception and symbolic numerical cognition is still unknown (Tsouli et al., 2022).

Here we ask whether numerosity-selective neural populations within the established network of numerosity maps are also involved in the processing of symbolic numbers. In our former study (Harvey et al., 2013), we did not find evidence to support the involvement of a map in the right superior parietal lobe (NPC1) in symbolic number processing. Here, we revisit this question with two conceptual advances. First, we evaluate the entire network of topographic maps, and we speculate that functional specialization of the maps differ (Tsouli et al., 2022), in particular for symbolic number processing. Second, we redesigned the stimulus and task. Specifically, we suspect that the lack of response to the presentation of numbers might have been because of the failure of perceiving the semantic meaning of the presented number symbols (i.e., the number concepts). In the Harvey et al. (2013) study, participants judged the color of the stimuli, but no number judgments were required. The magnitude information of nonsymbolic numerosity (e.g., a dot pattern of “••”) spontaneously emerges with a stimulus presentation in the visual format (Burr and Ross, 2008; Cicchini et al., 2016). However, this might not be the case for symbolic numbers (e.g., “2” or “two”), of which the physical appearance of a symbol bears no numerical information. Furthermore, attention appears necessary to numerosity perception (Burr et al., 2010; Anobile et al., 2012; Pomè et al., 2019; Cai et al., 2022). Thus, having a task that involves judgment of symbolic numbers will both focus attention on the number and force participants to process its magnitude information.

Using ultra-high field fMRI at 7 Tesla (Cai et al., 2021a), we recorded BOLD signals while participants were engaged in a symbolic number experiment with a number-detection task. We analyzed the neural responses to symbolic numbers using a GLM analysis throughout the cortex, and within the participant's numerosity maps. We applied a neural model-based analysis (i.e., pRF modeling) (Dumoulin and Wandell, 2008) to investigate whether numerosity-tuned neural populations at the numerosity maps are also tuned to symbolic numbers.

## Materials and Methods

### Participants

We present data from 7 participants (3 females, age range 24-48 years). Two were left-handed. The choice of the sample size is guided by a few considerations. Statistical power is determined by both number of trials per participant and number of participants (Baker et al., 2021). We focus on sufficient trials per participant rather than number of participants (sample size). For discussion in defense of small sample sizes (provided strong measurements), see Normand (2016) and Smith and Little (2018). Furthermore, this is in line with our previous studies where numerosity maps were identified with conventional localizer experiments (e.g., 8 participants in Harvey et al., 2013; Cai et al., 2021b; Tsouli et al., 2021; and 6 participants in Hofstetter et al., 2021). All the participants had normal or corrected-to-normal visual acuity. All were well educated, with good mathematical abilities. Written informed consent was obtained before every MRI session. All experimental procedures were approved by the ethics committee of Vrije University Amsterdam.

### Stimuli and experimental design

Visual stimuli were presented on a 69.84 × 39.29 cm LCD screen (Cambridge Research Systems) behind the MRI bore. Participants were required to lie still and view the display through a mirror attached to the head coil. The total distance from the attached mirror to the display screen was 220 cm. The display resolution was 1920 × 1080 pixels. A button box recorded behavioural responses. Visual stimuli were generated in MATLAB using PsychToolbox (Kleiner et al., 2007). A large diagonal cross composed of thin red lines was displayed consistently across the entire screen, serving as a fixation marker.

### Localizing nonsymbolic numerosity maps

**Figure 1.**
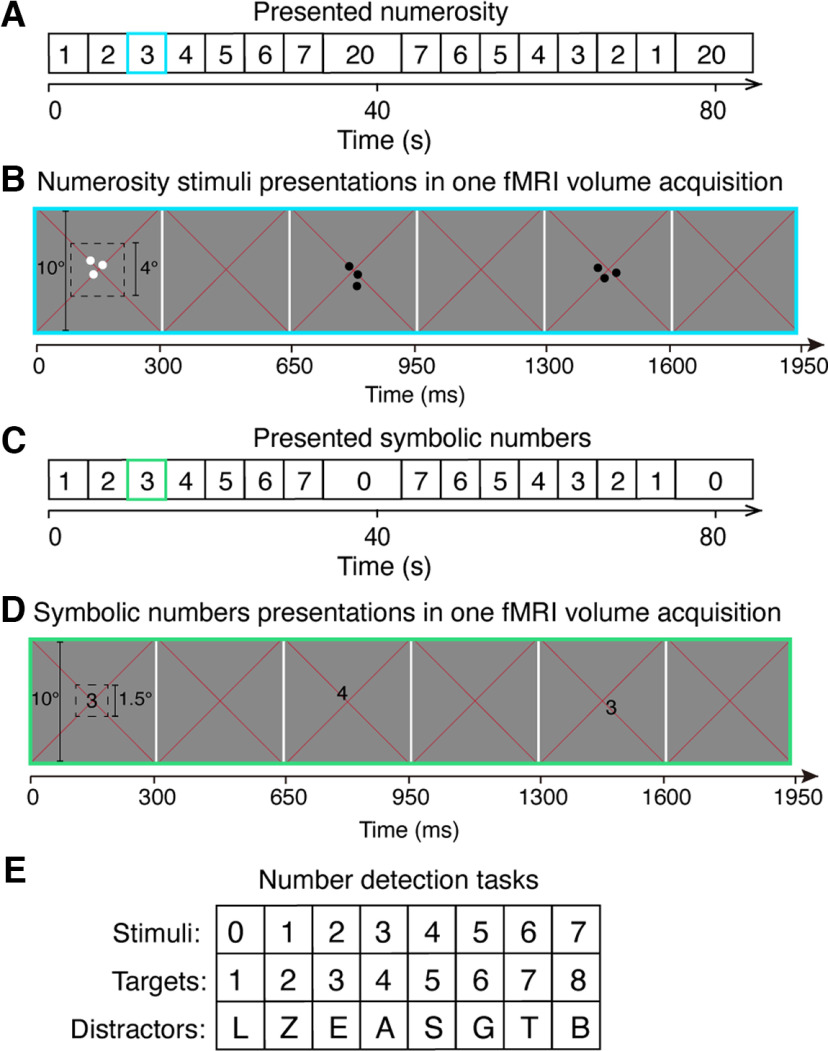
Illustration of experimental design and stimulus presentations. ***A***, The sequence of the presented nonsymbolic numerosity used to localize the numerosity maps. ***B***, Schematic representation of an example stimulus presentation in the numerosity maps localizer experiment (***A***, blue frame) within one fMRI volume acquisition (i.e., one TR). Each dot pattern was presented briefly (300 ms) at the central 4° of the visual field, followed by a 350 ms presentation of a gray background before a new random positioned dot pattern presentation. Each pattern of the same numerosity was repeatedly presented 6 times, corresponding to two TRs, before the numerosity changed. Participants fixated the red cross at the center and pressed a button when dots were shown in white rather than black. ***C***, The sequence of presented symbolic numbers. ***D***, Schematic representation of an example trial of symbolic number 3 in the symbolic experiment (***C***, green frame) within one TR. Each symbol was presented briefly (300 ms) at the central 1.5° of the visual field, with an interstimulus interval of 350 ms of a gray background. Each number was presented 6 times before moving to the next number. Participants fixated at the red cross and responded when the presented number increased in magnitude by 1 compared with the previous presented number. ***E***, Letters, similar in morphology to the target numbers, were used as distractors.

We first ran a localizer experiment to identify the numerosity maps of our participants in a separate session. We used the same study design as in previously studies (Harvey et al., 2013; Harvey and Dumoulin, 2017; Cai et al., 2021b; Hofstetter et al., 2021; Tsouli et al., 2021). Specifically, a sequence of numerosity stimuli consisting of 1-7 dots were first presented in ascending order, followed by a long period (15.6 s) where the baseline numerosity of 20 dots was presented, then followed by the same sequence in descending order and another identical baseline period (Fig. 1*A*). This sequence was repeated 4 times for each fMRI scan run.

Numerosity stimuli consisted of a group of dots with a constant total surface area presented in the central 4° (diameter) of the visual field. Dots were randomly positioned at each presentation so that each dot fell entirely within this area, to distribute contrast energy equally across the stimulus area for all numerosities. Each numerosity presentation that contained the same number of dots was placed in a new, random position, so no specific visual position was associated with any numerosity. To prevent perceptual grouping, individual items were distributed roughly homogeneously across the stimulus area. All of the numerosity stimuli were displayed as black or white dots on a gray background. Dot patterns were presented briefly (300 ms) to ensure that participants did not have time to count. A new random pattern was presented every 650 ms, with 350 ms presentation of a uniform gray background between dot pattern presentations. This was repeated 6 times, over 3900 ms, corresponding to two fMRI volume acquisitions (TR), before the numerosity changed (Fig. 1*B*). On 10% of numerosity presentations, the dots were shown in white instead of black. Participants were asked to fixate at the red cross in the center and press a button when whites dots were shown to ensure they were paying attention to the stimulus during fMRI acquisition. Participants responded to 90%-100% of the white dots presentations within each run. No numerosity judgements were required.

### Symbolic number experiment

We used a similar sequence as the numerosity stimulus sequence, but instead of dots we showed Arabic numbers from 1 to 7, and a baseline period using the number 0. The number 0 was used instead of 20 to keep all the stimuli as single-digit numbers (Fig. 1*C*) as 20 may be interpreted as containing two items (i.e., 2 and 0) (Bulthé et al., 2015). This stimulus sequence was presented in ascending (1-7), then descending order (7-1) followed by a baseline period, respectively. This sequence also repeated 4 times (cycles) for each functional run.

Symbolic number stimuli were randomly presented in the central 1.5° (diameter) of the visual field. Each number was presented briefly (300 ms) with an interstimulus interval of 350 ms of a uniform gray background between stimulus presentations. Each number was repeated 6 times, over 2 TRs before the number changed. Participants fixated the red cross and pressed a button when the number increased in magnitude by 1 compared with the previous presented number (Fig. 1*D*). A list of capitalized letters that were morphologically similar to the symbolic numbers from 1 to 8 were used as distractors: L, Z, E, A, S, G, T, B (Fig. 1*E*). All the numbers and letters were displayed in the font of Arial Unicode MS with the font size of 27. When the stimulus sequence was presented in an ascending order, there were always seven regular stimulus changes in symbolic number in one cycle that would require a response (embedded trials, e.g., when the stimulus changed from 1 to 2, 2 to 3, etc.). In addition to the embedded trials, at random points along the sequence, the stimuli presented was a number increased by 1 compared with the previous number (catch trials), or a letter that was morphologically similar to the target number (distract trials). The catch trials and distract trials correspond to 20% of the total number of stimulus presentation trials. A percentage of correct responses was calculated for the embedded trials and catch trials, respectively. Responses to the distractor letters or any trials other than the embedded trials and catch trials were counted as false alarms. Task performance was quantified using the discriminability index (*d'*) of the signal detection theory, which denotes participants' sensitivity to the targets (Green and Swets, 1966). We determined a response as a hit if it occurred within 2 s after a signal presentation; otherwise, it was classified as a false alarm.

### MRI acquisition and preprocessing

All MRI data were acquired using a Philips 7T scanner (Philips Medical Systems). MP2RAGE (Marques et al., 2010) T1 anatomic MRI data were acquired at the spatial resolution of 0.64 × 0.64 × 0.64 mm^3^ (resampled to 0.6 × 0.6 × 0.6 mm^3^ for following processing). TR = 6.2 ms, TE = 3 ms, flip angle (FA) = 5°. Functional T2*-weighted multiband (factor = 2) two-dimensional EPIs were acquired using a 32-channel head coil (Philip Nova Medical) at a resolution of 1.75 × 1.75 × 1.75 mm^3^. A full-brain-coverage (FOV = 106 × 112 × 236) covering 64 slices was used. TR = 1950 ms, TE = 25 ms, FA = 70°. Each functional scan run has 182 TRs (354.9 s in duration), of which the first six TRs were discarded to ensure the signal was at a steady state. Participants were scanned for eight functional runs in one session to localize the numerosity maps, except for Participant 4 who had nine runs. For the symbolic number experiment, each participant was scanned for two sessions on separate days, resulting in 15-17 functional runs in total.

T1 anatomic images were automatically segmented using cbs-tools (https://www.nitrc.org/projects/cbs-tools/) and then manually edited to minimize segmentation errors using ITK-SNAP (Yushkevich et al., 2006). This provides a highly accurate description of the cortical surface, an anatomic segmentation space used for analysis of cortical organization. The cortical surface was rendered as a smoothed 3D surface. Head movement and motion artifacts between and within functional images were measured and corrected for in AFNI (Cox and Hyde, 1997). Using Vistasoft (https://github.com/vistalab/vistasoft/wiki), the motion-corrected functional images were coregistered to the same anatomic space using the same transformation. The time-series data were aligned to the anatomy and then averaged. Data were imported to the anatomic segmentation space using trilinear interpolation. To increase signal strength, data from all recording sites (voxels) were collapsed onto the nearest point on the cortical surface layer. This formed a (folded) 2D representation of the gray matter nodes. pRF modeling and subsequent statistical analyses were performed at this space.

### pRF modeling of responses to nonsymbolic numerosity

pRF modeling was applied to estimate numerosity responses (Dumoulin and Wandell, 2008; Harvey et al., 2013). Briefly, a one-dimensional logarithmic Gaussian model was used to predict numerosity responses. This model describes tuning in logarithmic numerosity space using a Gaussian function characterized by preferred numerosity (mean of the Gaussian) and tuning width (SD of the Gaussian).

At each gray matter voxel, the pRF model is estimated based on the recorded signal and the predicted time course. A prediction of the neural response time course was produced by overlapping the stimulus (numerosity) at each time point with the Gaussian tuning function. By convolving this prediction with an HRF, a predicted response time course was generated. The pRF parameters for each voxel were chosen by those predicted fMRI time courses that bring the best agreement to the recorded signal, denoted as variance explained (*R*^2^). Last, participant-specific HRF parameters were estimated over the whole fMRI volume, and these parameters were used to refit the pRF.

**Figure 2.**
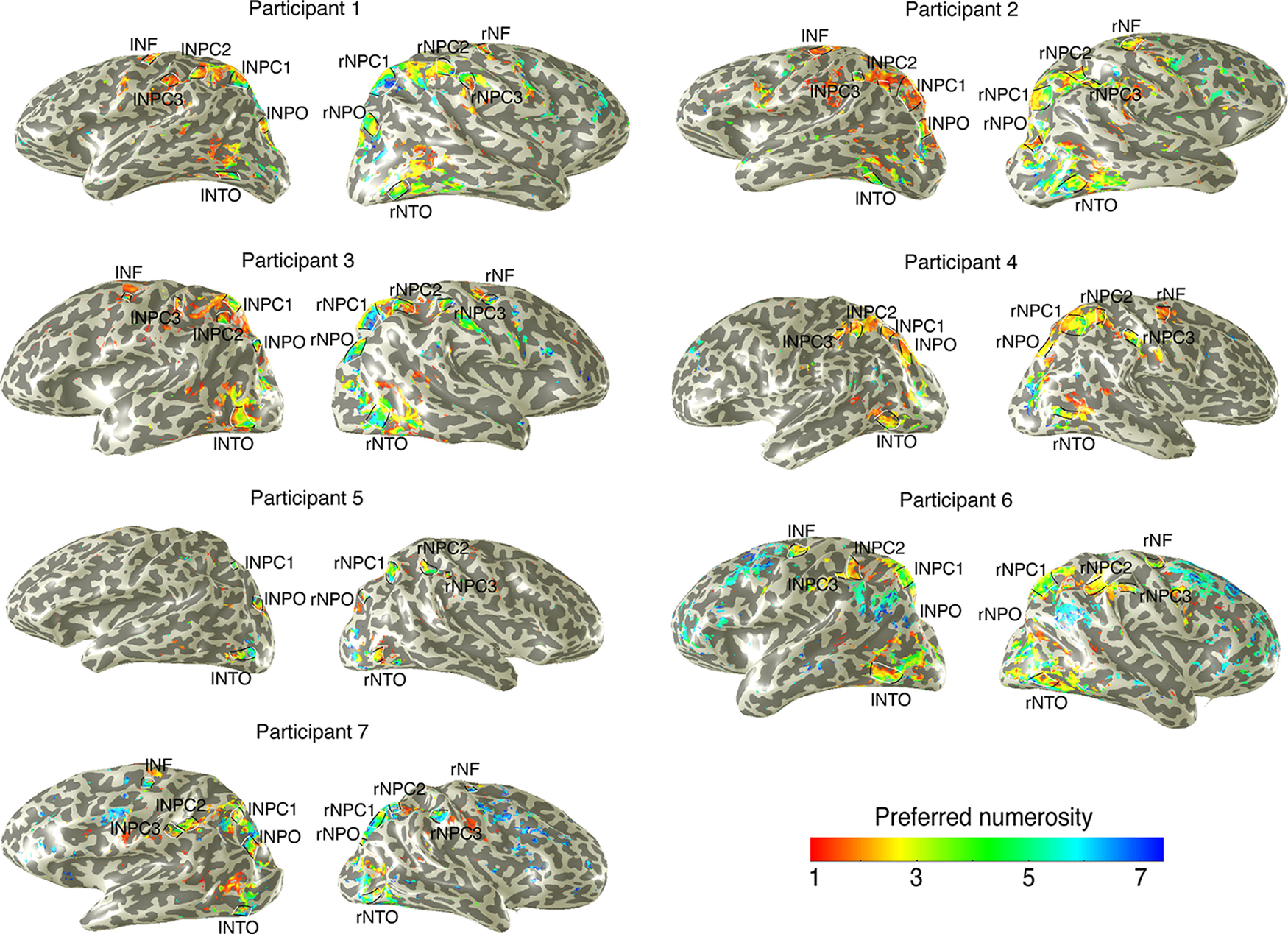
Cortical rendering of topographic numerosity maps of each participant. An example of preferred numerosity estimates in 1 participant for the left and right hemispheres. Black lines outline the edge borders of individual numerosity maps. White lines indicate the lowest and highest preferred numerosities in each map. The map of preferred numerosity estimates is thresholded at a variance explained of 30%. The numerosity maps served as independent ROIs in the symbolic number experiments.

The pRF fitting procedure allows preferred numerosity estimates outside the range of the presented stimuli, ensuring estimates within the stimulus range are not just the best of a limited set. We excluded from analysis any recording sites where the preferred numerosity was outside our presented range and the variance explained was <30%. We then projected the preferred numerosity of these recording sites on the unfolded cortical surface (Fig. 2).

### Definition of ROIs

We defined ROIs where the numerosity-selective neural populations are organized topographically similar to previously reported numerosity maps (Fig. 2) (Harvey and Dumoulin, 2017; Cai et al., 2021b; Hofstetter et al., 2021; Tsouli et al., 2021). In general, a network of six numerosity maps were defined on the left and right hemispheres, respectively. These maps lay in the temporal-occipital cortex (NTO), parietal-occipital cortex (NPO), parietal cortex (NPC1, NPC2, NPC3), and superior frontal cortex (NF). Within each ROI, we manually defined map borders on the lowest and highest preferred numerosities (white lines) and the map edges around the local regions with increase in model goodness of fit (black lines).

### Analysis of neural responses to symbolic numbers

First, we performed GLM analyses on the averaged functional data of the symbolic number experiment. To increase signal strength, we averaged the functional data across individual runs and collapsed data from the recording sites (voxels) across cortical depth onto a 2D cortical surface representation. At each cortical surface voxel (or vertex), we fitted a GLM with the main numbers (i.e., 1-7) presentation as a predictor and zero (0) as a baseline, resulting in a *t* value for each voxel. Then, we plotted these *t* values on the cortical surface above a statistically significant level (*p* < 0.05). Furthermore, to investigate whether numerosity-tuned neural populations also respond to symbolic numbers, we performed an ROI analysis focusing on the numerosity maps. The numerosity maps were defined by a separate localizer experiment using separate scans, separate stimuli on separate days (see Definition of ROIs). We averaged the time-series across all of the voxels within each numerosity map. We then fitted a GLM to the averaged time-series. We performed a repeated two-way ANOVA on the resulting *t* values of individual maps (ROI) across participants, followed by a *post hoc* analysis for multiple comparisons (JASP Team, 2020). Moreover, for each numerosity map, we performed a one-sample *t* test to test whether the neural responses to numerals in the map were significant (i.e., >0).

Next, we fitted pRF models, at the whole brain, to the averaged functional data (i.e., comprised of 16 functional scans) of the symbolic experiment. The pRF modeling procedure is similar to the numerosity modeling in the localizer experiment (see pRF modeling of responses to nonsymbolic numerosity). Nevertheless, in contrast to the (logarithmic) numerosity models, we fitted a Gaussian tuning function in linear space as symbolic numbers are more precisely and linearly represented (Verguts and Fias, 2004). In addition, to explore whether these responses are tuned responses to symbolic numbers, we calculated variance explained by pRF models that were fitted to the averaged data (i.e., comprised of 16 functional scans) of the symbolic number experiment at each numerosity map. Specifically, to increase signal strength, we first averaged time-series across all the voxels within each map. Then we calculated the variance of the averaged time-series explained by the prediction averaged by the corresponding voxel-wise predicted time-series. Next, we averaged the variance explained across participants at bilateral NTO maps, respectively (given that the GLM analyses showed that only NTO maps respond to symbolic numbers, we focused the analysis on these maps alone).

Moreover, we cross-validated the results of the pRF modeling and GLM analyses. We split the functional data of the symbolic number experiment into two halves based on odd or even runs. At each voxel, we extracted the response time-series from one half dataset and generated the predicted time-series from the other half dataset using the GLM or pRF models. Then, we fitted these two time-series to obtain a variance explained at each voxel. This procedure was repeated by swapping the two half datasets. The two resulting variances explained were averaged, yielding the cross-validated variance explained of the relevant voxel. We then quantified the proportion of recording sites where neural activity is better explained by tuning models rather than the GLM at bilateral NTO maps, respectively. Differences between the (cross-validated) variance explained of the pRF model and that of the GLM were calculated (i.e., ΔVE = VE__pRF_ – VE__GLM_). We performed a paired *t* test to compare the degree of the difference in the variance explained of the pRF model and GLM. A Wilcoxon's sign rank test was performed on the degree of difference to investigate whether the difference is significantly higher than zero.

Finally, we performed a Pearson correlation analysis between the preferred numerosity estimates and the preferred number estimates at the recording sites responding to both numerosity and symbolic numbers in the NTO map. Taking into account the functional resolution of the recording sites, the total number of data points (*n*) used to calculate correlation's probability was reduced by the factor by which functional voxels were upsampled onto the 2D cortical surface.

### Conversion to MNI coordinates

Our analyses were in individual participant space. To identify the location of the NTO map on an average brain, we converted these to MNI *x*, *y*, *z* coordinates. We first located at each individual participant's maps' centers on the cortical surface. We then transformed each participant's anatomic MRI data, together with these map center locations, into MNI averaged template space using MINC toolkit (Collins et al., 1994) (http://packages.bic.mni.mcgill.ca) and rigid alignment and linear scaling. We took the mean and SD of the resulting MNI coordinates of the NTO map across participants.

### Statistical analysis

All statistical analyses described in this paper were performed using the R2019b release of MATLB software (The MathWorks) unless indicated otherwise. Analyses were performed using repeated-measures ANOVA with *post hoc* analyses with Bonferroni corrected for multiple comparisons using JASP. A one-sample *t* test was performed to test statistical significance of stimulus-driven responses (*t* values) at group level. A paired *t* test was performed to compared the difference in (cross-validated) variance explained between the GLM and pRF models, and a Wilcoxon's sign rank test was performed to test the statistical significance. Results represent the mean ± SD as stated in the figure legends, unless indicated otherwise. Sample size and statistical tests are also reported in the figure notes.

## Results

### Participants engaged in the task

All the participants performed the task with a high percentage of correct responses, both of detecting changes in symbolic numbers as part of the stimulus sequence (embedded trials, mean ± SD: 93.6 ± 3.4%, *d'* = 4.1 ± 0.4) and random changes (catch trials, 83.3 ± 9.6%, *d'* = 3.7 ± 0.4). The percentage correct and *d'* suggest that participants were engaged in the task and processing the semantic meaning of the presented numbers (for the performance of individual participants, see Table 1).

**Table 1.** Task performance of the symbolic number experiment*^a^*

Participant	No. of runs	Target detection accuracy (%)		*d'*
		Catch trials	Embedded trials	
P1	15	79 ± 13	92 ± 6	3.4 ± 0.6
P2	16	70 ± 22	88 ± 6	3.7 ± 0.6
P3	17	95 ± 4	96 ± 3	4.2 ± 0.2
P4	16	88 ± 6	92 ± 5	3.7 ± 0.3
P5	16	78 ± 13	96 ± 4	3.5 ± 0.5
P6	16	77 ± 14	93 ± 6	3.4 ± 0.5
P7	16	95 ± 5	98 ± 3	4.3 ± 0.2
Average	16	84 ± 10	94 ± 3	3.7 ± 0.4

*^a^*Mean ± SD.

### Numerosity map NTO responds to symbolic numbers but not the other maps

**Figure 3.**
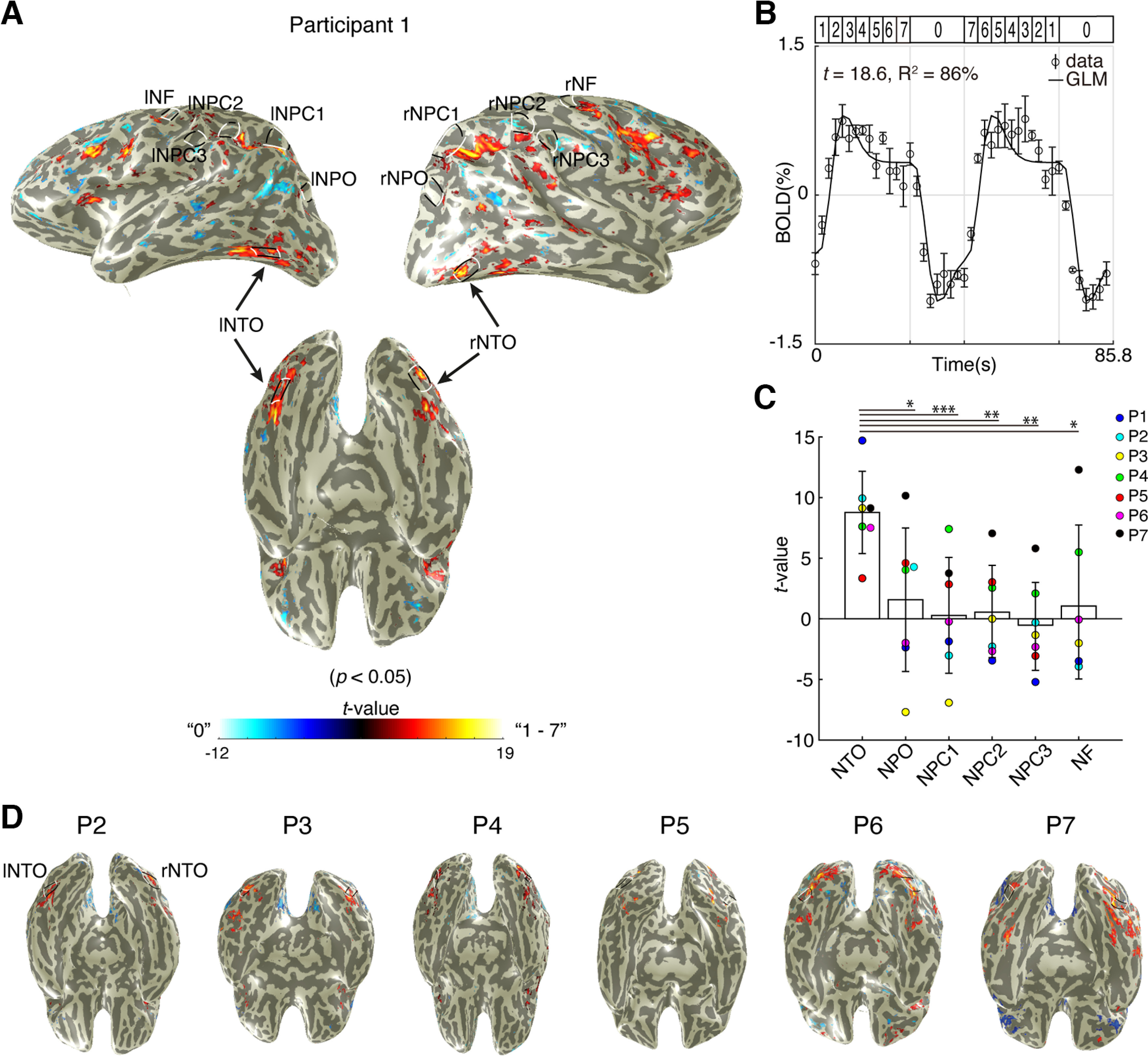
Stimulus-driven responses to symbolic numbers at numerosity maps. ***A***, The result of an example participant of the GLM analysis, which contrasted the responses to the number of 0 (blue) and 1-7 (yellow-red). Bottom, Ventral view of the cortical surface where the responses to symbolic numbers overlap with the NTO map. Only recording sites (i.e., voxels) with a *p* value <0.05 (equivalent to the variance explained [*R*^2^] by the GLM exceeding 30%) were projected on the cortical surface. ***B***, Response time-series to symbolic numbers at an example recording site extracted from the NTO map. The GLM prediction (solid line) captured 86% of the variance at this recording site with a corresponding *t* value of 18.6. Dots represent the averaged response amplitude. Error bars indicate SEM over repeated measures. ***C***, Averaged *t* values at individual maps across hemispheres and participants (*n* = 41). Repeated two-way ANOVA shows that responses at NTO are significantly higher than responses at the other maps (Bonferroni-corrected for multiple comparisons). Bars represent the mean. Error bars indicate SD of the mean. *p < 0.05. **p < 0.01. ***p < 0.001. ***D***, Ventral view of all other participants. Black and white lines outline the NTO maps.

Figure 3*A* shows the results of the GLM analysis of the main testing numbers of 1-7 (red) and the baseline number of 0 (blue). Most brain regions responding to symbolic numbers did not overlap with the location of the numerosity maps, except for NTO maps in the ventral stream of the bilateral temporal occipital cortices (Fig. 3*A*, bottom). To illustrate the responses to symbolic numbers, we extracted a time-series from an example recording site at the NTO map of Participant 1 (Fig. 3*B*). Responses were observed at the presentation of the main symbolic numbers (*t* = 18.6), and the GLM prediction captured most of the variance in the signal (*R*^2^ = 86%). Repeated two-way ANOVA with the factors of hemispheres and maps (see Materials and Methods) showed a significant effect of maps (*F*_(5,78)_ = 7.28, *p* < 0.001), but no significant effect of laterality (*F*_(1,78)_ = 0.142, *p* = 0.726) and no interaction effect (*F*_(5,78)_ = 1.124, *p* = 0.379). Based on these results, we averaged *t* values of individual maps across hemispheres for individual participants (Fig. 3*C*). *Post hoc* analysis showed that the NTO map exhibited significantly higher responses to symbolic numbers than other maps (Bonferroni-corrected for multiple comparisons, Fig. 3*C*). Furthermore, one-sample *t* test performed on the *t* values of each individual map across participants showed that only the NTO map exhibited responses significantly >0 (*t* = 6.12 × 10^−7^, *p* = 0.0005). These results indicate that neural populations at the NTO map not only respond to nonsymbolic numerosity but also to symbolic numbers. Ventral views of all other participants are shown in Figure 3*D*.

### Are responses at the NTO map also tuned to symbolic numbers?

**Figure 4.**
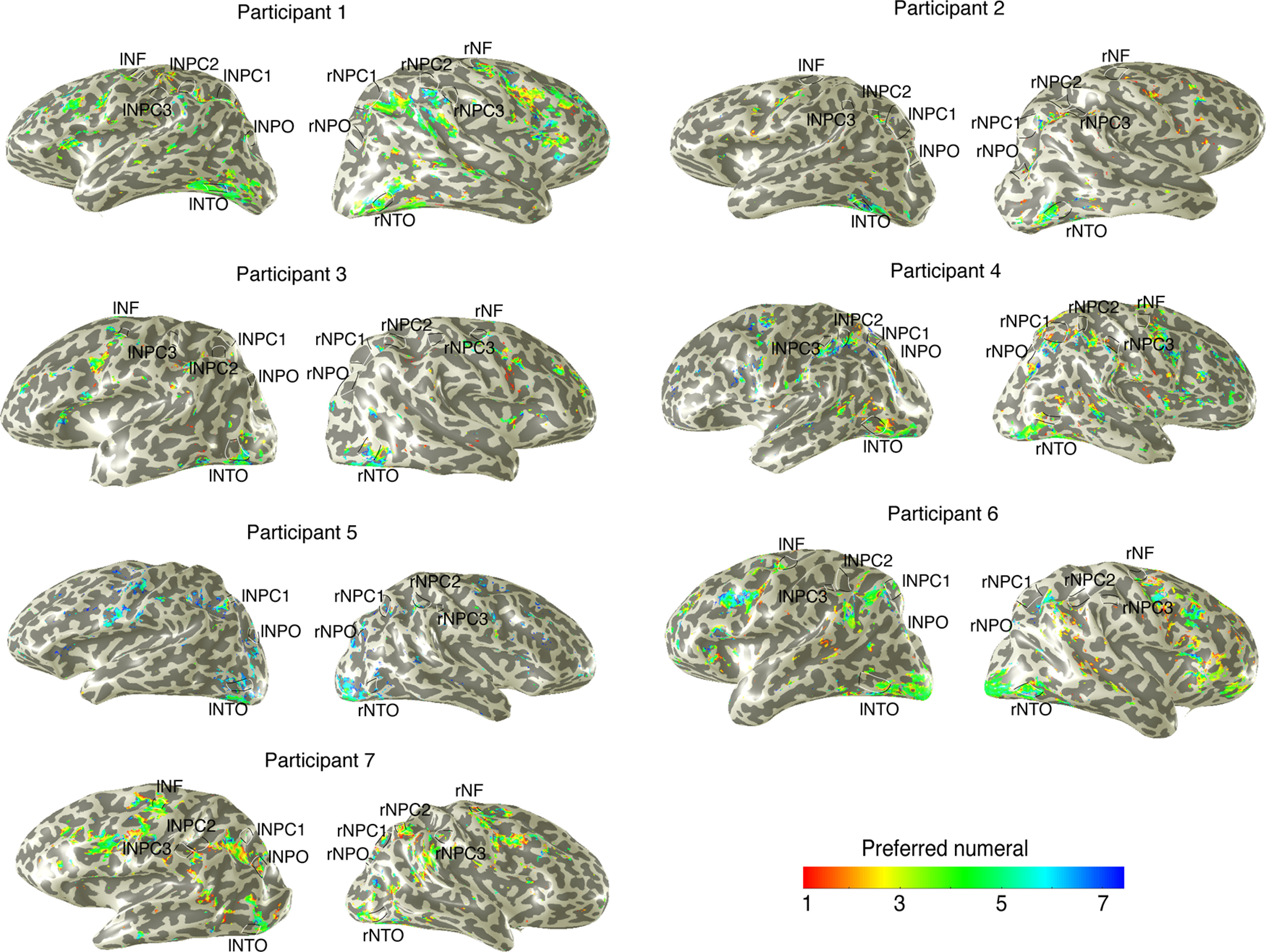
Tuned responses to symbolic numbers indicated by pRF modeling. Black and white lines indicate individual numerosity maps of each participant. Most tuned responses to symbolic numerals, defined by pRF models with a variance explained >30%, are found at the vicinity of the numerosity maps.

We projected on the cortical surface of voxels with >30% of their variance explained by the pRF models for symbolic numbers (Fig. 4). We found most of the responses at the vicinity of the numerosity maps (except for NTO maps), rather than overlap with the numerosity-tuned neural populations.

**Figure 5.**
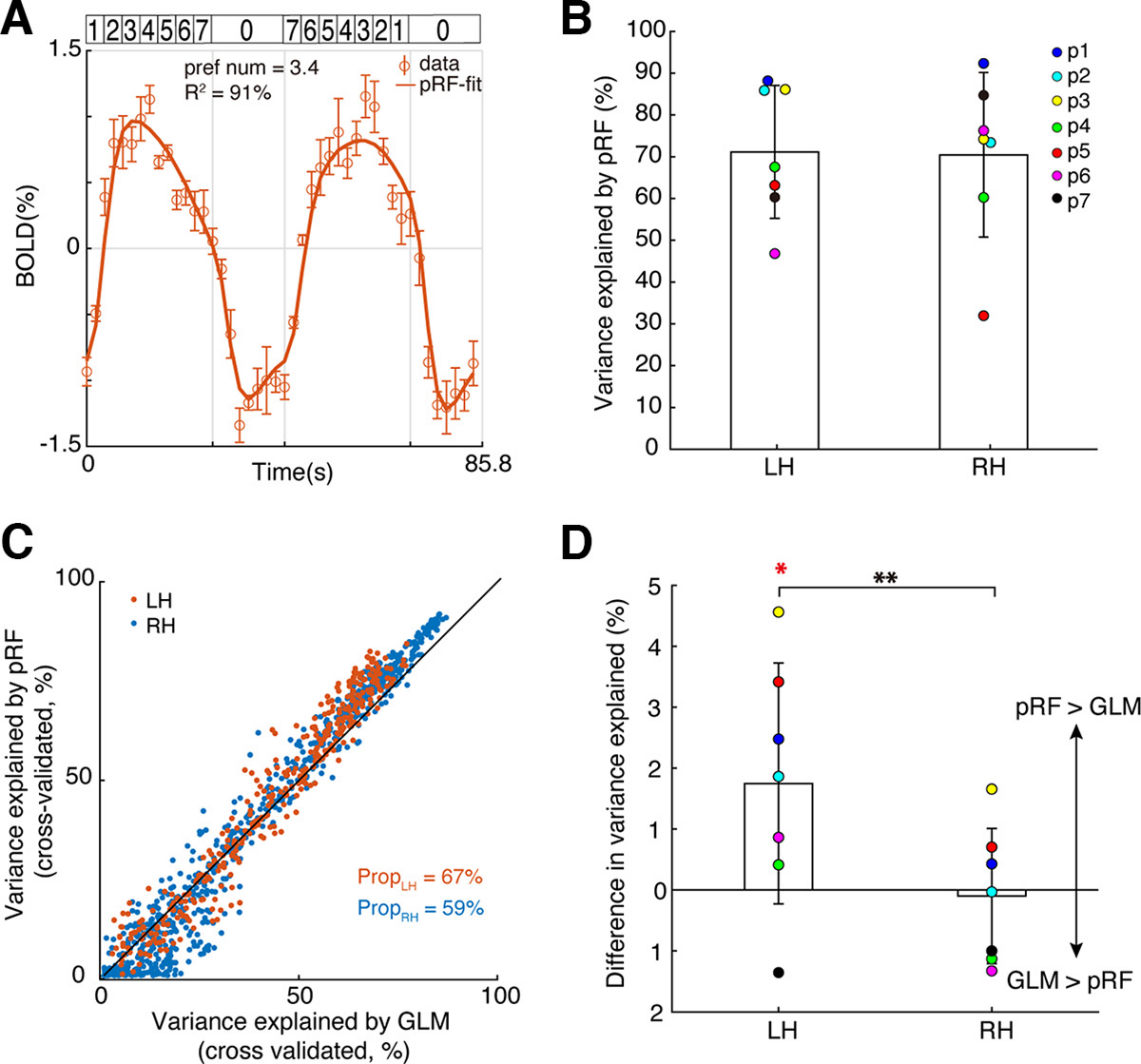
Numerosity-tuned neural populations at the left NTO map are tuned to symbolic numbers. ***A***, Response time-series of an example recording site at the NTO map of Participant 1 and the pRF model prediction. Dots represent the mean response amplitude. Error bars indicate SEM over repeated measures. The best fit neural model (solid line) captured >90% of the variance at this site. ***B***, Averaged variance explained of the pRF models that were fitted to the averaged data (i.e., comprised of 16 runs) of the symbolic number experiment, at bilateral NTO maps across participants (*N* = 7). ***C***, Cross-validated variance explained of the pRF models and GLM that were fitted to each half dataset (i.e., comprised of 8 runs) at all the recording sites within the NTO map of Participant 1. Black line indicates an equal variance explained by the GLM and pRF model. Texts indicate the proportion of recording sites where the pRF model fit the data better than the GLM. ***D***, Difference in variance explained at bilateral NTO maps derived by pRF and GLMs, averaged across participants. The NTO map in the left hemisphere shows significantly higher variance explained than zero (Wilcoxon's sign rank test, *p* = 0.0391), and significantly different from the variance explained of the right NTO map (paired *t* test, *p* = 0.004). LH, Left hemisphere; RH, right hemisphere. *p < 0.05. **p < 0.01.

Next, we asked whether the responses to symbolic numbers at the NTO maps show tuning characteristics which underlies perception (Tsouli et al., 2022), rather than untuned stimulus-driven responses. We found that the pRF models explained the data well and captured most of the response variance. Figure 5*A* shows a time-series of an example recording site at the NTO map of Participant 1 (open circles). The pRF model explained ∼90% of the variance at this site (solid line), and indicated that this recording site prefers the symbolic number of ∼3.4. Overall, the pRF models achieved considerable variance explained at bilateral NTO maps across all the participants (mean ± SD: *R*^2^ = 71 ± 16% in the left NTO map and 70 ± 20% in the right NTO map) (Fig. 5*B*).

We quantified the proportion of the recording sites where the tuning model explained more variance in the neural responses than the GLM (Fig. 5*C*,*D*). Overall, more than half of the recording sites at the NTO maps across participants and hemispheres showed higher (cross-validated) variance explained by the tuning model (mean ± SD: 56 ± 14% in the left NTO map and 52 ± 11% in the right NTO map). However, only the responses at the left NTO maps were significantly >0 (Wilcoxon sign rank test, *p* = 0.0391). Paired *t* test showed that the left NTO maps had a significantly higher variance explained by the tuning model than the right NTO maps (*t* = 4.53, *p* = 0.004).

**Figure 6.**
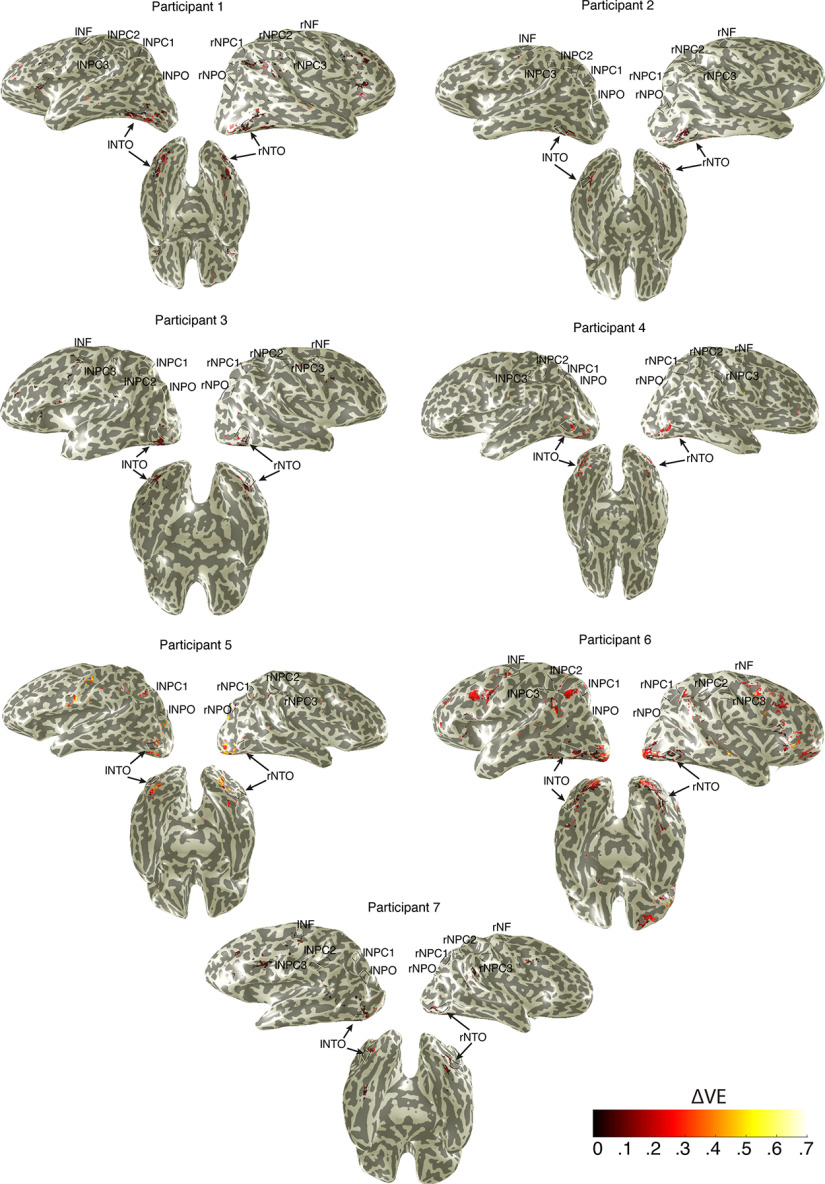
Subtraction between variance explained by pRF models and GLM highlights that the left NTO maps are also tuned to symbolic numbers. Black lines outline individual numerosity maps. This analysis was performed on those voxels with the variance explained by the pRF models or GLMs exceeding 30%. Only recording sites where the pRF variance explained were higher than the GLM variance explained were projected on the cortical surface.

We also projected on the cortical surface of voxels where the variance explained by the pRF models outperformed the GLM (i.e., ΔVE) (Fig. 6), which also highlights the left NTO map. These results suggest that numerosity-tuned neural populations at the left NTO map are also tuned to symbolic numbers.

We compared the preferred numerosity estimates and preferred number estimates of the neural populations in the left NTO map that responded to both stimuli. We did not find a significant correlation between these estimates (Pearson correlation coefficient, mean ± SD: *r* = 0.10 ± 0.2). These results suggest that the neural populations tuned to symbolic numbers in the left NTO map were distinct from the neural populations tuned to nonsymbolic numerosity.

Last, we investigated the NTO map in the context of other regions implicated in functional specializations in the ventral cortex, in particular the number form area (NFA). To identify the coordinates of the NTO map, we transformed the hemispheres of each participant into MNI space and averaged the coordinates across participants. Table 2 shows the coordinates at the center of the NTO map of individual participants in the current study and compared the averaged coordinates [values are given as mean (SD)] with those reported in our previous study (Harvey and Dumoulin, 2017). We then compared with the coordinates of the NFA previously reported in the inferior temporal gyrus and suggested to be specialized for Arabic numeral processing. Although we refrain from statistical analyses on these coordinates given all the differences in methods, we propose that NTO is close but distinct from the NFA.

**Table 2.** The MNI coordinates of the NTO map and the NFA*^a^*

Cortical regions	Reported studies	Left hemisphere			Right hemisphere		
		*x*	*y*	*z*	*x*	*y*	*z*
NTO	Current study-P1	−47	−60	−9	42	−71	−11
	Current study-P2	−35	−80	−8	35	−72	−8
	Current study-P3	−39	−70	−6	44	−71	−8
	Current study-P4	−42	−70	−1	40	−74	−5
	Current study-P5	−36	−71	−12	40	−81	−7
	Current study-P6	−42	−61	−15	41	−73	−14
	Current study-P7	−39	−56	−7	36	−77	−2
	Current study-average (*n* = 7)	−40 (4)	−67 (8)	−8 (4)	40 (3)	−74 (4)	−7 (4)
	Harvey and Dumoulin, 2017 (*n* = 5)	−42 (3)	−77 (3)	3 (8)	44 (7)	−75 (1)	−4 (3)
NFA	Shum et al., 2013 (*n* = 5)	—	—	—	51	−54	−24
	Abboud et al., 2015 (*n* = 9)	—	—	—	54	−45	−17
	Hermes et al., 2017 (*n* = 10)	—	—	—	57	−51	−17
	Yeo et al., 2017 (meta-analysis)	—	—	—	51	−49	−15
	Grotheer et al., 2016 (*n* = 24)	−60	−57	−17	61	−45	−17

*^a^n*, number of participants; —, no data.

## Discussion

Here we studied whether numerosity-tuned neural populations within a network of topographic maps respond to symbolic numbers. We used a number-detection task that requires participants' attention and forced processing of the semantic meaning of the presented Arabic numbers. Behavioral measures indicate that the participants indeed processed the semantic meaning of the numbers. We found that neural populations in the ventral temporal occipital cortex (NTO), but not the other numerosity maps, respond to symbolic numbers. The neural populations within the left NTO map were also found to be tuned to the presented numbers.

Previously, we explored whether numerosity-tuned neural populations also respond to symbolic numbers (Harvey et al., 2013). In that study, however, the focus was only on the superior parietal cortex (NPC1) and the experiment included a task that did not require participants to process the semantic meaning of the numbers. Here, we revisited this question with two differences: (1) we used a number-detection task that required the participants to process the semantic meaning of the presented numbers; and (2) we expanded our search to the entire network of numerosity maps, which covers the temporal, parietal, and frontal cortices (Harvey and Dumoulin, 2017; Cai et al., 2021b; Hofstetter et al., 2021; Tsouli et al., 2021). Analogous to the visual field maps, where multiple visual field maps are specialized for specific functions (Wandell et al., 2007), we hypothesized that different numerosity maps are also specialized for different functions (Tsouli et al., 2022). In line with our previous findings (Harvey et al., 2013), we did not observe responses to symbolic numbers at the NPC1 map in the parietal cortex. We did find responses to symbolic numbers in the NTO map at the ventral stream of the occipitotemporal region (VOT). These findings suggest that numerosity-tuned neural populations at the VOT region also play a role in symbolic number processing.

Moreover, the current study thoroughly explored all the known numerosity maps covering the whole human brain. A large body of studies have demonstrated that the human VOT region contains functional areas that exhibit strong selectivity for categories, such as faces (Kanwisher et al., 1997), bodies (Downing et al., 2001), word forms (Cohen et al., 2000), tools (Op de Beeck et al., 2019), and scenes (Epstein and Kanwisher, 1998). In addition, previous studies have localized an area in the inferior temporal gyri that shows selectivity to numbers symbols, namely, an NFA (Shum et al., 2013; Abboud et al., 2015; Hannagan et al., 2015; Grotheer et al., 2016; Hermes et al., 2017; Yeo et al., 2017). Thus, it seems likely that the neural populations tuned to symbolic numbers are located at the ventral stream that specialized for number symbols. However, we found that the anatomic locations of the NFA and the NTO map are not overlapping.

The results of this study shows that the neural population within the left NTO maps not only respond to symbolic numbers, but that these responses show tuning characteristics (i.e., they preferentially respond to a specific number). The preferred tuned responses to symbolic number, however, were uncorrelated with the preferred tuned responses to numerosity, suggesting that the underlying neural populations are distinct (Harvey et al., 2020; Hofstetter et al., 2021). This finding is in line with the observation that single-neuron recordings in the human medial temporal lobe not only showed neurons tuned to numerals, but also that the numeral-tuned and the numerosity-tuned neuronal populations were also distinct (Kutter et al., 2018). Of note, we applied a number-detection task to ensure that participants process the semantic meaning of the presented stimuli. This is different from the color-detection task used in the numerosity experiment. Thus, we cannot rule out the possibility that the tuning differences between numerosity and symbolic numbers at NTO maps might be because of the task differences. We did not find evidence for tuning to symbolic numbers in right NTO. This may be because of a genuine hemispheric difference but can also be attributed to methodological issues, such as the size of the map (Cai et al., 2021b) or to larger (scatter) tuning preferences of individual neurons, thereby blurring the tuning properties at the population level; that is, smaller size of the map or larger scatter may mask the tuning of single neurons at the population level.

Representations of numerosity in parietal and frontal brain regions are well investigated in both humans and nonhuman primates (Dehaene, 2003; Nieder and Dehaene, 2009; Harvey et al., 2013). Studies have demonstrated increased functional connectivity between ventral temporal regions with parietal and frontal regions during calculation (Park et al., 2012), and even in the blind (Abboud et al., 2015). Focusing on the numerosity maps, we found that only the NTO maps are also involved in symbolic number processing. However, it should be noted that other brain regions outside the numerosity network did show stimulus-driven responses during the symbolic numbers task, but little tuned responses to symbolic numbers. These results, however, were beyond the scope of this study as they did not overlap with the numerosity maps.

Representation of symbolic numbers is suggested to evolve from nonsymbolic numerosity representations (Dehaene and Cohen, 2007; Piazza et al., 2007). The high-level human numerical ability of processing numbers is believed to be linked to evolutionarily conserved numerosity representation during cognitive development (Halberda et al., 2008; Szkudlarek and Brannon, 2017). The finding of human number neurons also supports the hypothesis that symbolic number cognition is rooted in biologically determined mechanisms (Kutter et al., 2018). Our results of overlapping but distinct tuned neural populations to numbers and numerosity in the ventral temporal-occipital maps further support the link between nonsymbolic and symbolic numerical processing.

In conclusion, we found overlapping but distinct neural populations at the human temporal-occipital cortex (NTO maps) that respond to numerosity and to number stimuli. Moreover, the neural populations in the left temporal cortex (left NTO) were also tuned to symbolic numbers. These results support the hypothesis that numerosity perception is the precursor of the human-unique numerical abilities of processing number symbols.
